# Purinergic signalling and immune cells

**DOI:** 10.1007/s11302-014-9427-2

**Published:** 2014-10-29

**Authors:** Geoffrey Burnstock, Jean-Marie Boeynaems

**Affiliations:** 1 Autonomic Neuroscience Centre, University College Medical School, Rowland Hill Street, London, NW3 2PF UK; 2 Department of Pharmacology and Therapeutics, The University of Melbourne, Melbourne, Australia; 3 Institute of Interdisciplinary Research (IRIBHM), Université Libre de Bruxelles and Department of Laboratory Medicine, Erasme Hospital, 808, Route de Lennik, 1070 Brussels, Belgium

**Keywords:** ATP, UTP, Lymphocytes, Neutrophils, Mast cells, Microglia, Macrophages, Purinoceptors

## Abstract

This review article provides a historical perspective on the role of purinergic signalling in the regulation of various subsets of immune cells from early discoveries to current understanding. It is now recognised that adenosine 5′-triphosphate (ATP) and other nucleotides are released from cells following stress or injury. They can act on virtually all subsets of immune cells through a spectrum of P2X ligand-gated ion channels and G protein-coupled P2Y receptors. Furthermore, ATP is rapidly degraded into adenosine by ectonucleotidases such as CD39 and CD73, and adenosine exerts additional regulatory effects through its own receptors. The resulting effect ranges from stimulation to tolerance depending on the amount and time courses of nucleotides released, and the balance between ATP and adenosine. This review identifies the various receptors involved in the different subsets of immune cells and their effects on the function of these cells.

## Synopsis

ᅟ

## Introduction

ᅟ

## Purinergic signalling in the main subsets of immune cells

Polymorphonuclear leukocytes

*Neutrophils*

*Eosinophil*s

*Basophils*

*Mast cells*

*Section summary*

Monocytes, macrophages and microglia

*Monocytes*

*Macrophages*

*Microglia*

*Section summary*

Dendritic cells

*P1 receptors*

*P2 receptors*

*Section summary*

Lymphocytes

*T and B lymphocytes*

*Natural killer (NK and NKT) cells*

*Section summary*

## Concluding remarks

ᅟ

## Introduction

Although nucleotides, such as adenosine 5′-triphosphate (ATP) and uridine 5′-triphosphate (UTP), are mainly intracellular, they are released into the extracellular fluids by various mechanisms. One of them is cell damage and death: both necrotic and apoptotic cells release ATP and other nucleotides that thus constitute “danger signals” or damage associated molecular pattern [1–3]. In the absence of cell death, they are also released in response to various types of stress: mechanical stimulation (stretch, shear stress) [4], hypoxia or pathogen invasion [5, 6]. Specific mechanisms of release include: exocytosis of secretory granules, vesicular transport [7, 8] and membrane channels, such as ATP-binding cassette transporters, pannexins [9–11] and connexins [12]. In particular, nucleotides are released by exocytosis during platelet aggregation and synaptic transmission. For many years, cells of the immune system were not considered to be innervated, but there is increasing recognition that nerves can influence the immune system and the field of neuroimmunology is growing rapidly [13–15].

Once in the extracellular fluids, nucleotides are rapidly degraded by a variety of ectonucleotidases [16], such as the ENTPDases, like CD39 that degrades ATP into adenosine 5′-diphosphate (ADP) and ADP into adenosine monophosphate (AMP) and CD73/5′-nucleotidase that converts AMP into adenosine. Receptors for extracellular nucleotides and their degradation products such as adenosine have been progressively characterized. Subdivision of purinergic receptors between P1 (adenosine) and P2 (ATP, ADP) was proposed by Burnstock in 1978 [17]. A further subdivision of P2 receptors between P2Y and P2X was made in 1985 [18]. It is now well established that signalling by extracellular nucleotides is mediated by these two families of receptors, the molecular structure of which has been characterized: P2Y receptors are metabotropic G protein-coupled while P2X receptors are oligomeric ion channels.

Numerous reviews on various aspects of purinergic signalling in the immune system are available (Table 1). In the history and development of knowledge about purinergic signalling, early workers focussed on adenosine, while those concerned with ATP rarely referred to adenosine. This is obviously an inadequate approach since the effects of ATP and adenosine, its breakdown product that is rapidly produced by ectonucleotidases, are intimately related. In this review, purinergic signalling in immune cells will be covered in a comprehensive and historical way, following the increase in knowledge from the early discoveries to current understanding. The review will consider the major subsets of immune cells and, for each of them, address the mechanisms of nucleotides release and adenosine generation, as well as the repertoire of functional P1 and P2 receptors that they express.

**Table 1:** Reviews on the role of purinergic signalling in the immune system

General reviews on purinergic signalling in the immune system [474, 537–543]
Immune regulation by extracellular nucleotides [544–551]
Immune regulation by adenosine [552–563]
Ectonucleotidases and immune responses [426, 564–566]
Purinergic signalling in neutrophils [567–569]
Purinergic signalling in eosinophils [570]
Purinergic signalling in mast cells [571, 572]
Purinergic signalling in monocytes [573]
Purinergic signalling in macrophages [574]
Purinergic signalling in microglia [344, 575, 576]
Purinergic signalling in dendritic cells [577, 578]
Purinergic signalling in lymphocytes [579–586]
P2X_7_ receptors and immune cells [383, 503, 587–589]
P2X_7_ receptors, macrophage function and bacteria [590–594]

## Purinergic signalling in the main subsets of immune cells

### Polymorphonuclear leukocytes

#### Neutrophils

##### P1 receptors

Ectoenzymes that hydrolyse ATP have been observed on guinea pig polymorphonuclear leukocytes [19–21]. In particular, both CD39 and CD73 ectonucleotidases are present on neutrophil membranes [22]. Furthermore, neutrophils express mRNA for A_1_, A_2A_, A_2B_ and A_3_ receptors [23], but the mRNA for A_2A_ and A_3_ receptors are the most abundant [24]. Adenosine was shown to be a physiological modulator inhibiting the generation of superoxide (O_2_^−^) anion by neutrophils via A_2_ receptors [25–29]. Not surprisingly, dipyridamole, which prevents the uptake of adenosine, thereby increasing extracellular levels, inhibits O_2_^−^ generation by neutrophils [30]. Adenosine also inhibited the degranulation induced by the chemotactic peptide N-formyl-methionyl-leucyl-phenylalanine (fMLP) [28], phagocytosis [31] and the bactericidal function of neutrophils [32]. It was proposed that the inhibitory actions of adenosine on neutrophils were due to calcium entry blockade [33–35]. Adenosine inhibited neutrophil respiratory bursts in association with an increase in cyclic AMP (cAMP) and reduction in [Ca^2+^]_i_ [36, 37]. Occupancy of A_2A_ receptors by adenosine inhibits fMLP-induced neutrophil activation via cAMP and protein kinase A regulated events [38]. Caffeine intake results in increase in cAMP accumulation and decrease in O_2_^−^ anion production in human neutrophils, mediated by A_2A_ receptors [39]. Activation of A_2A_ receptors also inhibited the expression and release of various cytokines and chemokines after lipopolysaccharide (LPS) stimulation of neutrophils [40]. But other studies showed that both the A_2B_ and the A_3_ receptors can also play a role in these inhibitory actions. Tumour necrosis factor-α (TNF-α) production by neutrophils following renal ischemia-reperfusion was increased in A_2B_-deficient mice [41]. Activation of A_2B_ receptors also inhibited fMLP-induced O_2_^−^ production [42]. The A_3_ receptor is also involved in the inhibition of O_2_^−^ production [43] and of degranulation [44]. Adenosine downregulated ligand-stimulated leukotriene B_4_ biosynthesis in neutrophil suspensions [45], but it potentiated neutrophil cyclooxygenase-2 via A_2A_ receptors [46].

**Fig. 1:**
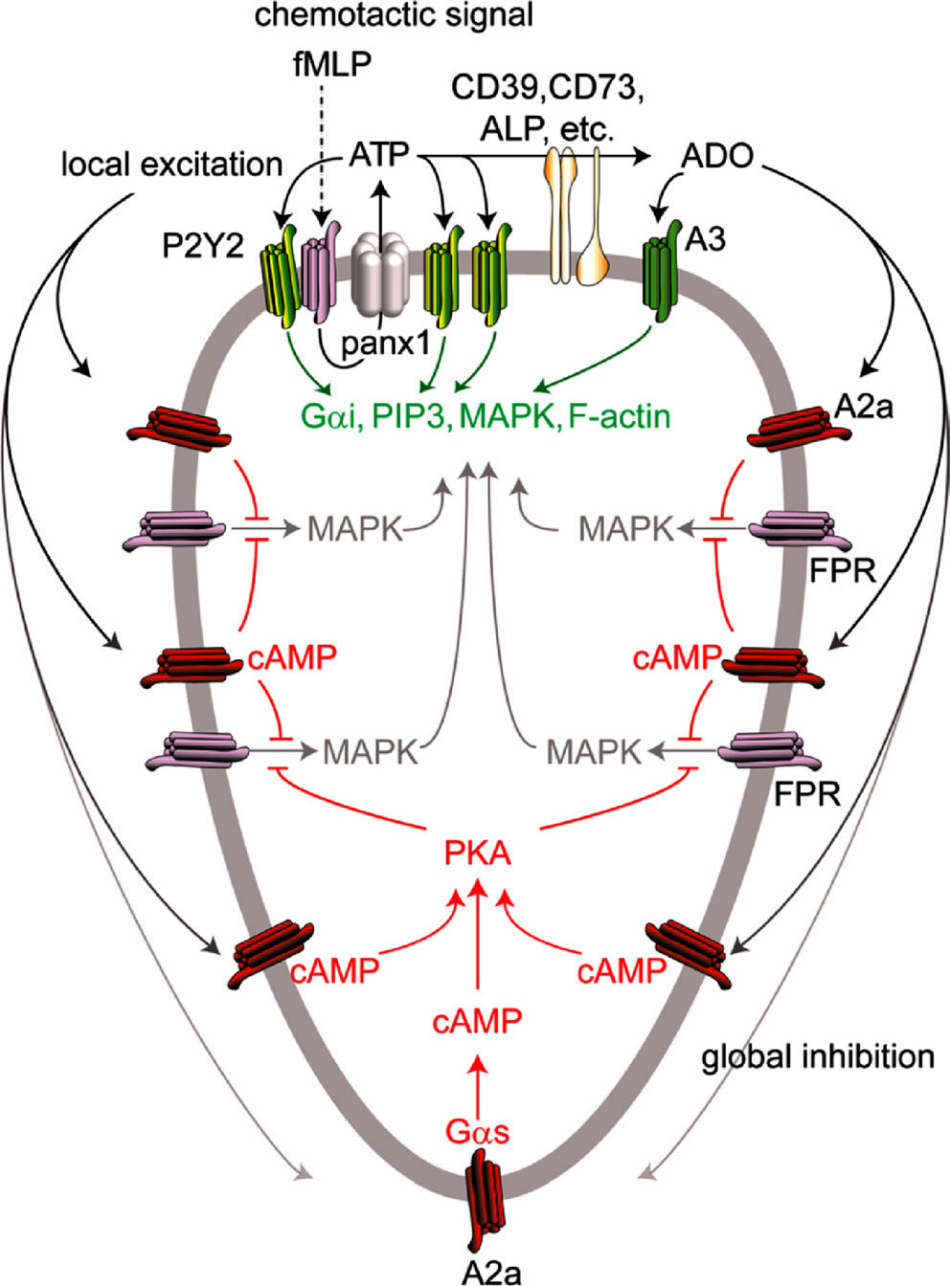
Proposed model of neutrophil chemotaxis. As previously reported, stimulation of chemoattractant receptors induces local release of ATP through PANX1 channels at the site that first encounters the chemoattractant. Autocrine feedback via P2Y_2_ receptors amplifies the chemotactic signal and triggers cell polarization, whereby cells assume an elongated shape, and PANX1, CD39 (NTPDase1) and A_3_ adenosine receptors accumulate at the leading edge. In the current study, we found that A_2A_ receptors are translocated from the leading edge toward the back of polarized neutrophils and that inhibitory signaling via A_2A_ receptor-dependent cAMP accumulation inhibits excitatory chemotactic signalling by blocking FPR-dependent ERK and p38 MAPK activation globally with the exception of the leading edge. *ALP* alkaline phosphatase, *ADO* adenosine, *PIP3* phosphatidylinositol (3,4,5)-triphosphate. (Reproduced from [55], with permission from the American Society for Biochemistry and Molecular Biology)

In contrast, there are discrepant reports concerning the action of adenosine on neutrophil chemotaxis. It was claimed in 1982 that adenosine had no effect on the chemotaxis of neutrophils, although it did enhance the inhibition of chemotaxis by 3-deaza-(±)aristeromycin [47]. However, it was reported later that adenosine promotes neutrophil chemotaxis [48], perhaps via A_1_ receptors [49]. It was shown recently that the recruitment of neutrophils and other leukocytes in the lung during influenza infection is reduced in A_1_-deficient mice [50]. In contrast, LPS-induced recruitment of neutrophils in the lung was increased in A_2A_-deficient mice and experiments with chimeric mice revealed that this involves a direct inhibitory effect of the A_2A_ receptor in myeloid cells [51]. Similar results were obtained in A_2B_-deficient mice [52]. Interestingly, in A_2A_-deficient mice, neutrophils were increased in the alveolar space [51], whereas they were increased in the interstitium of A_2B_-deficient mice [52]. Chen et al. [24] showed that adenosine stimulates neutrophil migration and amplifies the action of chemotactic signals through A_3_ receptors that are recruited to the leading edge (see Fig. 1). In A_3_-deficient mice, the recruitment of neutrophils was reduced in the lung during sepsis [53] and in the colon after induction of colitis by dextran sulphate [54]. Interestingly neutrophil chemotaxis requires excitatory signals at the front and inhibitory signals at the back of cells. This inhibitory signal at the back might be mediated by adenosine acting on A_2A_ receptors [55] (Fig. 1).

There is evidence that adenosine can modulate the interaction of neutrophils with pathogens. A_3_ receptors aggregate in highly polarised immunomodulatory microdomains of human neutrophil membranes. They promote the formation of filipodia-like projections (cytonemes) that can extend up to 100 μm to tether pathogens. Exposure to bacteria or an A_3_ agonist stimulates the formation of these projections and bacterial phagocytosis, whereas an A_3_ antagonist inhibits cytoneme formation [56].

Neutrophil adherence to endothelium was enhanced via A_1_ receptors and inhibited via A_2_ receptors [57, 58]. It is now believed that adenosine generated from ATP by CD39 and CD73 on the vascular surface functions as an anti-adhesive signal for neutrophil binding to microvascular endothelia through activation of neutrophil adenosine A_2A_ and A_2B_ receptors [59]. Activation of A_2A_ receptors also inhibits expression of α4/β1 integrin on human neutrophils [60]. Human neutrophils activated by fMLP increased the number of cell surface β_2_ integrins on endothelial cells and induced the shedding of L-selectin. These effects were inhibited by adenosine, most likely via the A_2A_ receptor [61]. A_2_ receptor activation inhibited neutrophil injury to coronary endothelium [62]. Adenosine also acts on endothelial receptors, thereby promoting vascular barrier function, providing a mechanism to dampen vascular leak syndrome during neutrophil–endothelial interactions [63] and regulating neutrophil chemotaxis [64]. Exposure of human endothelial cells to hypoxia/re-oxygenation caused increased neutrophil adhesion, an effect prevented by adenosine [65]. Adenosine also reduced the stimulatory effect of neutrophils on tissue factor-dependent coagulant activity of endothelial cells as a result of the inhibition of neutrophil adhesion to endothelial cells mediated by A_2_ receptors [66].

Adenosine might also play a role in the regulation of neutrophil number. Synergistic effects of granulocyte colony-stimulating factor and dipyridamole increased neutrophil production in mice [67]. Both effects were inhibited by adenosine deaminase (ADA). Theophylline has an immunomodulatory action on neutrophil apoptosis via A_2A_ receptor antagonism [68].

The expression of adenosine receptors on neutrophils can be modulated in pathological conditions and following various interventions. A_2A_ receptors on freshly isolated human neutrophils are upregulated after stimulation by LPS or TNF-α, and this may represent a feedback mechanism to control inflammation [69]. A_2B_ receptor activity in neutrophils is reduced in patients with systemic sclerosis [70]. A 4.6-fold decrease in adenosine-mediated inhibition of neutrophils from patients with septic shock was reported [71]. Hypertonic saline upregulates A_3_ receptor expression on activated neutrophils and increases acute lung injury after sepsis [72]. Alterations in the functional expression of both A_2A_ and A_3_ receptors in human neutrophils treated with pulsing electromagnetic fields have been reported [73, 74].

##### P2 receptors

ATP induces an increase in [Ca^2+^]_i_ in human [75] and mouse [76] neutrophils. ATP and UTP, acting via P_2U_ (i.e. P2Y_2_ and/or P2Y_4_) receptors, coupled to the inositol 1,4,5-trisphosphate pathway and increased [Ca^2+^]_i_ [37]. This was associated with a priming of neutrophils for enhanced O_2_^−^ generation when stimulated by other agonists [37, 77, 78]. The release of Ca^2+^ from thapsigargin-sensitive intracellular stores is essential for this nucleotide-induced priming in human neutrophils [79], indicating mediation via P2Y receptors. Enhanced O_2_^−^ responses of rat neutrophils stimulated by formyl chemotactic peptide were evoked by ATP and ADP, whereas adenosine and AMP were inhibitory [80–82]. ATP and UTP also stimulated granule secretion from human neutrophils [83, 84] and potentiated the secretion induced by chemotactic peptides [78]. They also induced neutrophil aggregation [78, 85].

Human neutrophils release ATP from the leading edge of the cell surface to amplify chemotaxic signals and direct cell orientation by feedback via P2Y_2_ receptors (Fig. 1) [24, 55, 86]. The importance of this mechanism in pathology is demonstrated by studies showing that the infiltration of neutrophils in the smoke-injured lung [87] and in the liver damaged by toxic agents [88] is decreased in P2Y_2_ knockout (^−/−^) mice. Chen et al. [24] also showed that neutrophil ectonucleotidases hydrolyze ATP to adenosine, which, via A_3_ receptors, also promoted cell migration (Fig. 1). In agreement with this concept, both P2Y_2_ and A_3_ receptors control the recruitment of neutrophils to the lungs in a mouse model of sepsis [53]. Neutrophil chemotaxis requires excitatory signals at the front and inhibitory signals at the back of cells that regulate cell migration. P2Y_2_ receptors, as well as A_3_ receptors, were shown to contribute to excitatory signals at the front, while adenosine acting on A_2A_ receptors contributed to the inhibitory signal at the back [55] (Fig. 1).

The P2Y_14_ receptor was shown to be functionally expressed on human neutrophils [89], and uridine-diphosphate (UDP)-sugars promoted Rho-mediated signalling and chemotaxis in human neutrophils [90], which was blocked by a P2Y_14_ antagonist [91].

Neutrophil apoptosis induced by ATP was inhibited by P2Y_11_ receptor activation, and it was suggested that targeting of P2Y_11_ receptors could retain the immune functions of neutrophils and reduce the injurious effects of increased neutrophil longevity during inflammation [92]. A later paper showed that P2Y_11_ receptors mediate ATP-enhanced chemotactic responses of rat neutrophils [93].

RT-PCR and Northern blot analysis revealed the presence of P2X_7_ receptors on neutrophils and 2′ (3′)-O-(4-benzoylbenzoyl) ATP (BzATP), a potent P2X_7_ receptor agonist, stimulated production of O_2_^−^ [23, 94]. A role of P2X_7_ in protection against neutrophil apoptosis has been reported [95, 96]. Neutrophil accumulation in the skin during croton oil-induced irritant contact dermatitis was reduced in P2X_7_-deficient mice [97]. However, it was claimed more recently that human neutrophils do not express P2X_7_ receptors [98]. In an RT-PCR study of human neutrophils, mRNA for P2X_1_ was strongly expressed, while mRNA for P2X_4_ and P2X_5_ was weakly expressed and P2X_7_ mRNA was not detected [99]. P2X_1_ receptors mediate neutrophil chemotaxis via Rho kinase activation [100]. A study using P2X_1_ receptor knockout mice led to the conclusion that P2X_1_ receptors play a protective role in endotoxaemia by negatively regulating systemic neutrophil activation, thereby limiting the oxidative response, coagulation, and organ damage [101].

#### Eosinophils

##### P1 receptors

A_3_ receptors were identified on human eosinophils and their activation led to increased [Ca^2+^]_i_ [102]. However, the role of adenosine and A_3_ receptor signalling on this cell type remains controversial with both pro- and anti-inflammatory activities of adenosine being reported. Adenosine was shown to potentiate the production of O_2_^−^ by guinea pig pulmonary eosinophils [103]. However, an inhibition of degranulation and O_2_^−^ anion release from human eosinophils was observed later and shown to be mediated by A_3_ receptors [104]. In human eosinophils, adenosine inhibits chemotaxis via the A_3_ receptor [105, 106], whereas a stimulatory effect has been observed in eosinophils of ADA-deficient mice [107].

##### P2 receptors

**Fig. 2:**
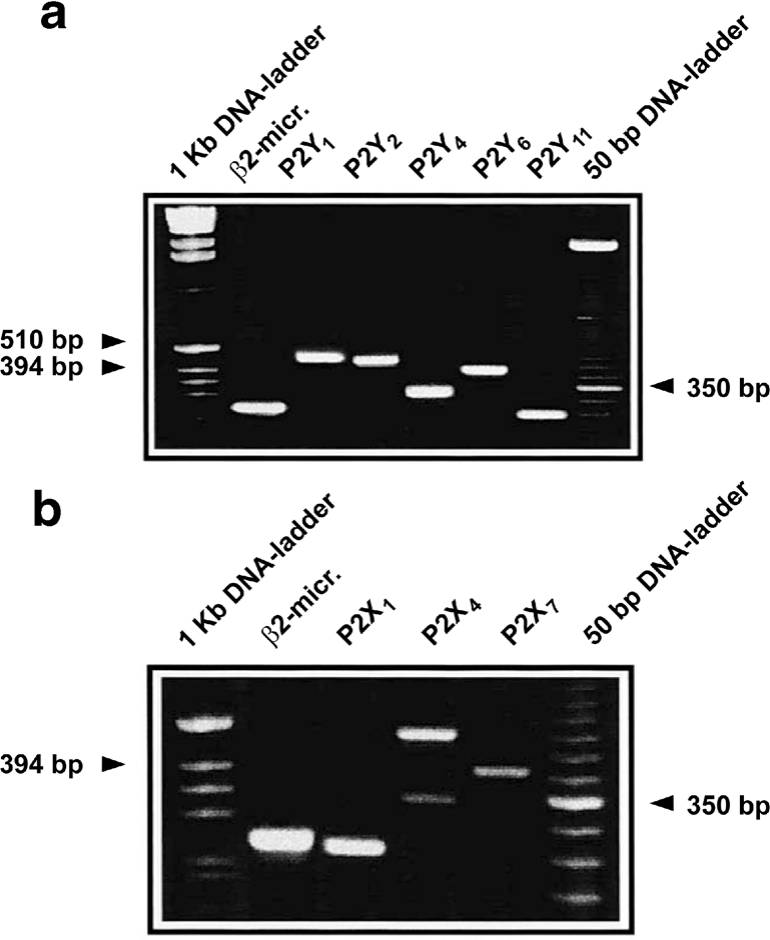
P2 receptors expressed by human eosinophils. **a** P2Y receptors. **b** P2X receptors. (Reproduced from [110], with permission from Elsevier)

Nucleotides were shown to stimulate human eosinophils, and it was suggested that since ATP is released from autonomic nerves and activated platelets, it could modulate the migration and other activities of eosinophils in vivo [76]. Thrombin-stimulated platelets secrete ATP, a chemotactic factor that attracts eosinophils [108]. ATP was shown to be a potent activator of eosinophils, suggesting a role for ATP in the pathogenesis of eosinophilic inflammation as an activator of pro-inflammatory effector functions [109]. Expression of P2Y_1_, P2Y_2_, P2Y_4_, P2Y_6_, P2Y_11_ and P2X_1_, P2X_4_, P2X_5_ and P2X_7_ receptor mRNA has been observed in human eosinophils (see Fig. 2) [99, 110]. It was also shown in this paper that purinoceptors mediate increase in [Ca^2+^]_i_ and the production of reactive oxygen intermediates. The functional characterization of P2Y and P2X receptors on human eosinophils was undertaken, and it was shown that UTP and ATP had a greater stimulatory effect on the production of reactive oxygen metabolites, actin polymerization and chemotaxis than the selective P2X receptor agonists α,β-methylene ATP and BzATP, suggesting a predominant role of P2Y receptors [111]. However, P2Y and P2X agonists had similar effects regarding intracellular calcium transients and the adhesion molecule CD11b. In a study of human eosinophils, ATP was shown to trigger secretion of eosinophil cationic protein, probably via P2Y_2_ receptors, while ATP induced interleukin (IL)-8, probably via P2Y_6_, P2X_1_ and P2X_7_ receptors [112]. Autocrine release of ATP and P2 receptors, presumably P2Y_2_, were shown to play a pivotal role in human eosinophil degranulation and production of pro-inflammatory cytokines in response to the endogenous danger signal, crystalline uric acid [113]. Human eosinophils respond also to ADP via P2Y_12_ receptors to elicit eosinophil secretion of peroxidase [114]. The use of knockout mice has allowed us to demonstrate the crucial role of P2Y_2_ receptors in the accumulation of eosinophils in the lungs during allergic inflammation. This involves both a direct chemotactic effect of ATP mediated by the eosinophil P2Y_2_ receptor [115] and an indirect effect on endothelial cells, where ATP via P2Y_2_ stimulates the expression of VCAM-1 that mediates eosinophil adherence and infiltration, and its soluble form that is chemotactic for eosinophils [116].

#### Basophils

##### P1 receptors

It was reported that human basophils have a receptor for adenosine that mediates inhibition of immunoglobulin (Ig)E-mediated histamine release [117, 118]. In later papers, it was shown that the inhibitory effect of adenosine is mediated by an A_2_ receptor and cAMP increase [119–121].

##### P2 receptors

Activation of permeabilised rat basophilic leukaemia cells (RBL-2H3) by adenosine-5′-O-(3-thio)triphosphate led to secretion of allergic and inflammatory mediators [122]. In a recent paper, it was shown that degranulation and histamine release from human basophils, associated with type 1 allergy, was evoked by UTP and particularly UDP, suggesting mediation by P2Y_2_ and/or P2Y_4_ and P2Y_6_ receptors [123].

#### Mast cells

##### P1 receptors

Potentiation of A23187 calcium ionophore-induced mast cell release of histamine by adenosine was initially reported [124]. Anti-IgE-induced release of histamine from mast cells was also enhanced by adenosine [125–130], as was β-hexosaminidase release from bone marrow-derived mast cells [131]. Histamine release from human adenoidal mast cells induced by concanavalin A or acetylcholine was also enhanced by adenosine [132]. Although other mechanisms have been proposed [133, 134], the potentiation of histamine release by adenosine appears to be mediated by A_3_ receptors, since it was mimicked by selective A_3_ agonists [135, 136] and abolished in A_3_-deficient mice [137]. Furthermore, it was shown that the increase of cutaneous vascular permeability and extravasation of plasma proteins in response to adenosine was abolished in mast cell-deficient mice as well as in A_3_-deficient mice [138]. Similarly, adenosine-induced bronchoconstriction was attenuated in mast cell-deficient and A_3_-deficient mice [139].

The response of human lung mast cells to adenosine was biphasic: low concentrations of adenosine potentiated release of histamine, while high concentrations elicited inhibition [140]. Adenosine also inhibited IgE-dependent degranulation of human skin mast cells via A_2A_ receptors [141]. Both A_2A_ and A_2B_ receptors were identified on mouse bone marrow-derived mast cells [142]. Using knockout mice, it was demonstrated that the inhibition of mast cell degranulation by adenosine in mediated by the A_2B_ receptor, while the combined action of A_2B_ and A_2A_ receptors is responsible for the inhibition of cytokine production [143]. However, the role of adenosine receptors in mast cell regulation is more complex, since the stimulatory effect of adenosine on the release of angiogenic factors such as vascular endothelial growth factor (VEGF) was shown to involve a cooperation between A_2B_ and A_3_ receptors [144, 145]. Furthermore, in umbilical cord blood-derived mast cells, IL-4 increased the potentiating effect of adenosine on degranulation via an upregulation of A_2B_ receptors, whereas these receptors were shown previously to be inhibitory in murine mast cells [146].

##### P2 receptors

ATP was reported early to evoke calcium-dependent histamine release with degranulation of rat mast cells [147–152]. It was suggested that the source of ATP may be innervating nerve fibres [153, 154], as discussed below. A correlation was shown between the ATP levels in rat peritoneal mast cells and histamine released by the anaphylactic reaction and compound 48/80 [155, 156]. ATP was shown to induce cytokine expression and apoptosis via P2X_7_ receptors on murine mast cells [157], supporting the earlier recognition of ATP-induced pore formation in rat peritoneal mast cells [158]. Interestingly, colitis was improved in mast cell-deficient mice as well as in those mice reconstituted with P2X_7_^−/−^ mast cells, showing the role of mast cell activation by ATP via the P2X_7_ receptor in intestinal inflammation [159]. ATP-induced cytokine and chemokine expression could also be mediated by P2X_1_ and P2X_3_ receptors on murine mast cells [160]. Functional expression of P2X_1_, P2X_4_ and P2X_7_ receptors in human lung mast cells was presented [161]. Mast cells are a major source of protein arginine deiminase, and it was shown that ATP induced protein arginine deiminase 2-dependent citrullination in mast cells via P2X_7_ receptors [162].

G protein-coupled P2Y receptors were also shown to mediate mast cell activation [163, 164]. UDP-glucose acting via P2Y_14_ receptors was shown to be a mediator of mast cell degranulation and considered as a potential therapeutic target for allergic conditions [165]. In a recent paper, all P2Y receptor subtypes were shown to be expressed in variable levels by human LAD2 mast cells [166]. Although P2Y_4_ and P2Y_11_ receptors were highly expressed, they did not appear to play a major role in degranulation, whereas P2Y_14_ receptors did.

**Fig. 3:**
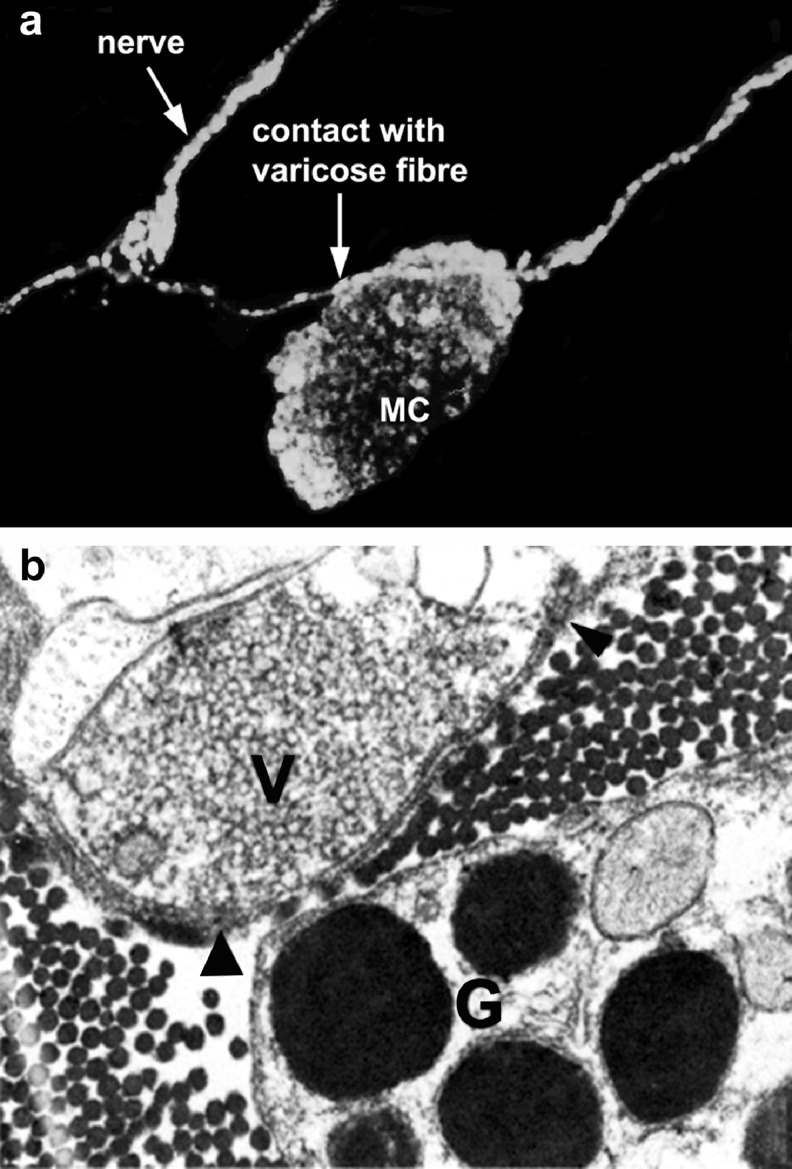
**a** Close apposition between rat mast cell protease 1 immunoreactive and calcitonin gene-related peptide immunoreactive nerve fibres observed by confocal microscopy. **b** Ultrathin section of rabbit middle cerebral artery showing granular cells (*G*) separated by a distance of less than 200 nm. *V* varicosities; *arrowheads* basement membranes. Magnification, ×29374. (**a** Reproduced from [176] and **b** from [173], with permission from Elsevier)

Autonomic nerves as well as sensory-motor nerves innervate immune cells and release ATP as a cotransmitter in close vicinity of immune cells [167]. Indeed in accordance with the definition of the autonomic neuroeffector junction, close contact of nerve varicosities with effector cells in effect constitutes innervation, albeit of a transient nature [168, 169]. Mast cells were the first claimed to be innervated [170]. Antidromic stimulation of sensory nerves increased degranulation of mast cells in the skin, and this effect was mimicked by ATP [171]. Close opposition of nerve varicosities containing small and large vesicles and mast cells in the mucosa of intestine was shown with electron microscopy [154, 172] and also in cerebral blood vessels [173] (Fig. 3). Synovial mast cell activity that contributes to inflammation in joints was shown to be influenced by both unmyelinated afferent and sympathetic efferent nerves [174]. Sympathetic and trigeminal sensory nerve fibres influence rat dural mast cells and have been shown to play a role in the pathophysiology of vascular headache [175]. Functional interactions between sensory nerves and mast cells of the dura mater have been described in both normal and in inflammatory conditions [176]. Vagus nerve stimulation modulates histamine content in mast cells in the rat jejunal mucosa [177]. From a study of co-cultures of nerves and mast cells, it was concluded that ATP released from activated mast cells was an important mediator to activate nerves [178, 179]. While substance P released from nerves activated mast cells, ATP released from mast cells in response to anti-IgE antibody activated superior cervical ganglia neurons. Few investigations have been carried out about the influence of nerves on non-mast cell immune cells, but evidence has been presented that nerve fibres form close relationships with other immune cells, such as eosinophils [180], macrophages [181], and T and B lymphocytes [182–184].

#### Section summary

Adenosine and ATP have opposite effects on O_2_^−^ generation and other functions of neutrophils: adenosine has an inhibitory effect, mediated mainly by A_2A_ and A_2B_ receptors, while ATP has a potentiating effect. On the other hand, ATP and adenosine cooperate to amplify the migration of neutrophils induced by chemotactic signals: this involves a stimulatory effect mediated by P2Y_2_ and A_3_ receptors expressed at the front of the neutrophils and an inhibitory effect of A_2A_ receptors expressed at the back of the cells.

ATP via P2Y_2_ receptors also plays an important role in the migration of eosinophils and their accumulation in the lungs during allergic inflammation. Adenosine exerts a dual effect on mast cell degranulation: stimulation through A_3_ receptors and inhibition via A_2A_ and A_2B_ receptors.

### Monocytes, macrophages and microglia

#### Monocytes

##### P1 receptors

Adenosine was initially reported to inhibit the production of the second complement component (C2) of human monocytes [185], and this effect was later shown to be mediated by A_2_ receptors [186]. Subsequently, it was also shown that A_1_ receptors were expressed on cultured human monocytes and rheumatoid synovial fluid mononuclear phagocytes [187]. Enhancement of Fcγ receptor-mediated phagocytosis was induced via A_1_ receptors, while A_2_ receptors mediated reduction of Fcγ phagocytosis in cultured monocytes. TNF-α production in human monocytes was inhibited by P1 receptor agonists [188]. Both A_2A_ and A_2B_ receptors were shown to be involved in the inhibition of TNF-α production [189]. Activation of A_2A_ receptors also inhibited IL-12 and stimulated IL-10 production by human monocytes [190, 191]. These actions may contribute to suppression of Th1 responses. However, the effect of P1 receptor agonists on cytokine release from human mononuclear cells was shown to depend on the specific Toll-like receptor (TLR) subtype used for stimulation: the A_2A_ agonist CGS21680 inhibited TLR4-mediated TNF-α release, but potentiated TLR3- and TLR5-mediated IL-6 release [192]. Activation of A_2A_ receptors also inhibited LPS-induced IL-18 production, expression of adhesion molecules and production of TNF-α, in human monocytes [193, 194]. Activation of A_1_ receptors promoted multinucleated giant cell formation by human monocytes [195]. Adenosine analogues were shown to produce apoptosis of human mononuclear cells via A_2A_ and A_3_ receptors [196].

##### P2 receptors

**Fig. 4:**
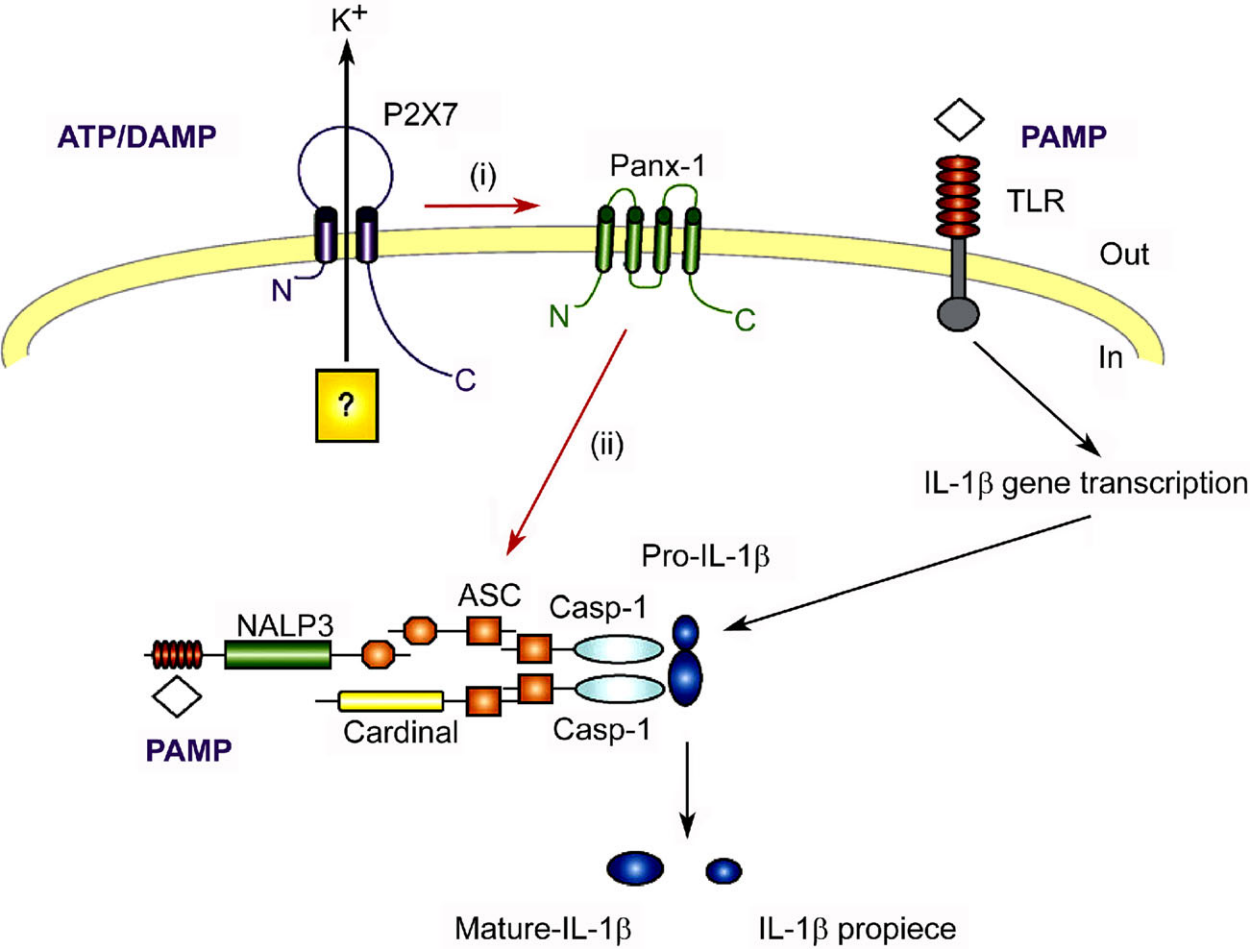
Hypothetical sequence of events leading to P2X_7_ receptor and pannexin 1 (panx-1)-mediated inflammasome activation. Pathogen-associated molecular patterns (*PAMPs*) bind to Toll-like receptors (TLRs) and drive interleukin (IL)-1β gene expression and accumulation of the pro-cytokine. Extracellular ATP binds to the P2X_7_ receptor and triggers K^+^ efflux and panx-1 activation. The functional significance of K^+^ efflux is unknown, although it might facilitate or even precipitate inflammasome activation. Likewise, the mechanism of panx-1 activation by the P2X_7_ receptor is unknown. Panx-1 in turn activates the inflammasome. Data suggest that the ion-carrying activity of panx-1 is unnecessary for inflammasome activation. The activated inflammasome then cleaves pro-IL-1β. Thus, stimulation of the inflammasome by extracellular ATP can be split into two steps: (a) recruitment and activation of panx-1 by the P2X_7_ receptor and (b) activation of the inflammasome by panx-1. Colour coding: *white* PAMP, *red* TLR, *green* NALP3 inflammasome, *orange* protein–protein interaction domains, further subdivided into *orange square*, *ASC* apoptosis-associated speck-like protein containing a caspase-recruitment domain and *orange octagon*, pyrin domain; *yellow* FIIND domain, *light blue* caspase domain (Casp-1), *dark blue* biologically active IL-1β and IL-1β propiece, *violet* P2X_7_ receptor, *light green* panx-1. (Reproduced from [210], with permission from Elsevier)

ATP and ADP were initially shown to increase [Ca^2+^]_i_ in monocytes and to regulate the activity of adhesion receptors CD11b/CD18 [197]. ATP and ADP activated human promonocytic U-937 cells apparently via different P2 receptor subtypes [198]. mRNA for P2X_7_ and P2Y_2_ receptors was shown to be expressed by human THP-1 monocytic cells and monocytes, and the presence of these receptors was supported by pharmacological data [199–201]. P2X_7_ receptor expression in THP-1 monocytes was positively modulated by pro-inflammatory stimuli and negatively modulated by cAMP, a classic anti-inflammatory second messenger [202]. P2X_7_ receptors mediated ATP-induced IL-1β release from human and canine monocytes [203–205], an effect requiring priming by LPS [206]. This mechanism plays a major role in the physiological control of IL-1β secretion by monocytes. Indeed microbial components acting on different pathogen-sensing receptors, as well as the danger signals uric acid and C3a, induced the activation of human monocytes and their secretion of IL-1β and IL-18 through a process involving, as an initial event, the release of ATP [207–209]. This was followed by the autocrine stimulation of P2X_7_ receptors and inflammasome activation [210] (Fig. 4). Indeed, IL-1β secretion was inhibited by apyrase as well as by P2X_7_ antagonists. Additional evidence in favour of the involvement of P2X_7_ was the observation that the P2X_7_ receptor polymorphism Glu496Ala, which is associated with a loss of function, impaired ATP-induced IL-1β release from human monocytes [211].

ATP was initially described as a chemoattractant for monocytes [212, 213]. More recently apoptotic thymocytes were found to release nucleotides leading to the recruitment of monocytes [3]. This release is mediated by pannexin 1 channels, as demonstrated by the use of pharmacological inhibitors and small interfering RNA (siRNA), and involves the activation of pannexin 1 by caspases [9]. Monocyte recruitment by apoptotic cells supernatants, demonstrated *inter alia* in the murine air-pouch model, was decreased in P2Y_2_^−/−^ mice, leading to impaired clearance of the apoptotic cells. These data clearly identify ATP as a find-me signal acting through the P2Y_2_ receptor that recruits monocytes in order to clear apoptotic cells.

Other effects of extracellular nucleotides on monocytes include increased surface expression of Mac-1 integrin [214], secretion of IL-8 that might involve P2Y_2_ and P2Y_6_ receptors [215, 216], inhibition of soluble HLA-G secretion [217], secretion of VEGF [218] and modulation of phagocytosis [219]. These last 3 effects involve P2X_7_ receptors. In human monocytes, ATP was reported to increase cAMP via the P2Y_11_ receptor, and thereby to inhibit proinflammatory cytokines production and to increase the release of IL-10 [213].

#### Macrophages

##### P1 receptors

Chemotaxis and lysosomal secretion were shown to be inhibited by adenosine and analogues in the mouse macrophage cell line RAW 264 or murine peritoneal macrophages [220, 221]. Adenosine was reported to inhibit TNF-α expression, induced by LPS in the mouse macrophage cell lines J774.1 [222] and RAW264.7 [223], whereas it potentiated nitric oxide synthase (NOS) expression induced by LPS in RAW 264.7 mouse macrophages [224, 225]. Interferon (IFN)-γ upregulated A_2B_ receptor expression in macrophages [226], while TNF-α or LPS induced A_2A_ expression via nuclear factor-κB, as part of a feedback mechanism for macrophage deactivation [227, 228]. TNF-α release from macrophages was inhibited by adenosine via A_2A_ and A_2B_ receptors [229–232] and IL-10 production was augmented by adenosine acting through A_2B_ [233] or A_2A_ [234, 235] receptors. Interestingly, it was shown that pro-inflammatory macrophages (M1 cells that release TNF-α) have a low expression of ecto-nucleotidases and rate of ATP hydrolysis as compared to anti-inflammatory macrophages (M2 cells that release IL-10) [236]. A_2A_ receptors also upregulated the expression of peroxisome proliferator-activated receptors [237] and hypoxia-inducible factor 1 [238]; this could contribute to the anti-inflammatory and tissue-protecting action of adenosine. A_2A_ receptors mediated upregulation of vascular endothelial growth factor expression in murine [239] and human [240] macrophages. On the other hand, activation of A_3_ receptors stimulates matrix metalloproteinase-9 secretion by macrophages [241], and glucocorticoids promote survival of macrophages through stimulation of A_3_ receptors [242].

##### P2 receptors

Early reports showed that ATP permeabilised the plasma membrane to fluorescent dyes [243, 244], promoted cation fluxes [245–247], increased [Ca^2+^]_i_, induced a respiratory burst and O_2_^−^ generation [248, 249], inhibited phagocytosis [250] and induced cytotoxicity [251] and cell lysis [252] in a variety of macrophage populations. ATP was also shown to stimulate phosphoinositides hydrolysis and eicosanoid synthesis in mouse peritoneal macrophages [253]. Oxidized ATP (oxATP) was shown to irreversibly inhibit the permeabilization of the plasma membrane, but not the fast mobilization of Ca^2+^ induced by ATP in macrophages, supporting the expression of P2X_7_, then called P2_Z_, receptors in the J774 macrophage cell line [254]. P2X_7_ receptors were also shown to be expressed by BAC1.2F5 mouse macrophages, mediating both pore-forming and phospholipase (PL)-D activity [255], and in human monocyte-derived macrophages [256, 257].

Later studies demonstrated the involvement of the P2X_7_ receptor in several responses of macrophages to danger, in particular the proinflammatory response mediated by IL-1β secretion, bacterial killing and the associated macrophage death. ATP was shown to promote the maturation and release of IL-1β from macrophages [258, 259], via P2X_7_ receptors [260, 261]. ATP-induced secretion of IL-1β was abolished in macrophages from P2X_7_-deficient mice and involved inflammasome assembly and caspase-1 activation [262–264]. Activation of the inflammasome and release of IL-1β in macrophages dying through autophagy [265] or stimulated by serum amyloid A [266] involved the release of ATP and the activation of P2X_7_. P2X_7_^−/−^ mice showed increased survival after lung adenoviral infection, resulting from a decreased production of IL-1β by macrophages [264]. These mice were also protected against smoke-induced lung inflammation and emphysema, as a result of decreased activation of lung macrophages [267].

P2X_7_-mediated ATP-induced killing of mycobacteria by human macrophages was initially reported in 1997 [268]. This seminal observation was later confirmed in numerous studies. Mycobacterial killing involved phagosome–lysosome fusion [269] that was induced by the rise of Ca^2+^ and the activation of PLD resulting from P2X_7_ activation [270]. It was decreased in macrophages from P2X_7_^−/−^ mice [271]. Infection by mycobacteria upregulated the expression of P2X_7_ and its activation by ATP not only enhanced intracellular bacterial killing but also induced the apoptosis of macrophages [272] or autophagy [273]. This dual response was missing in macrophages from P2X_7_^−/−^ mice [271]. ATP-induced bacterial killing was abrogated in macrophages from individuals homozygous for a loss of function P2X_7_ polymorphism [274] and reduced by 50 % in heterozygous subjects [275]. Additional polymorphisms leading to similar consequences were described later [276]. Furthermore, the pattern of gene expression in response to ATP was different in patients with tuberculosis and controls, suggesting that a defective function of P2X_7_ might lead to the development of tuberculosis [277]. Infection by parasites, such as *Leishmania amazonensis* [278, 279] and *Toxoplasma gondii* [280, 281], also increased the expression of P2X_7_ that mediated a dual response of parasite killing and macrophage apoptosis.

The P2X_7_ receptor is also involved in various additional responses of macrophages. ATP released by LPS increased NOS expression and NO production in RAW 264.7 macrophages via P2X_7_ receptors [282–288]. The P2X_7_ receptor was also associated with the generation of reactive oxygen species (ROS) [289–291] and leukotriene B_4_ [279, 292]. Activation of P2X_7_ receptors on macrophages induces the activation and release of tissue factor and thus favours thrombosis [293, 294]. Phagocytosis of nonopsonised beads and heat-killed bacteria was increased by P2X_7_ over-expression, showing that it can behave as a scavenger receptor, but this effect was inhibited by ATP [219, 295]. Loss of function polymorphisms of P2X_7_ and P2X_4_ receptors were associated with reduced phagocytosis and were overrepresented in patients with macular degeneration [296]. P2X_7_ receptors play a role in the generation of macrophage-derived giant cells, a hallmark of chronic inflammation [297]. Spontaneous cell fusion was indeed described in macrophage cultures expressing high levels of the P2X_7_ receptors [298]. Furthermore, the formation of multinucleated giant cells was inhibited by P2X_7_ antagonists and in macrophages from P2X_7_-deficient mice [299, 300].

Despite the dominant role of P2X_7_ in macrophages, evidence has accumulated to support the role of additional receptors. Multiple P2X and P2Y receptor subtypes were identified in mouse J774, spleen and peritoneal macrophages [301]. In an extensive study, mRNA for P2X_1_, P2X_4_, P2X_5_, P2X_7_, P2Y_2_, P2Y_4_, P2Y_6_, P2Y_11_, P2Y_13_ and P2Y_14_ receptors were all expressed by human alveolar macrophages [302]. It was suggested that other P2X receptor subtypes, in addition to P2X_7_ receptors, were involved in the ATP-mediated current in human macrophages [303]. In particular it was shown that a small slowly-desensitising ATP-induced current was abolished in P2X_4_^−/−^ mice [304]. This P2X_4_ response might contribute to the P2X_7_-induced cell death that was reduced by siRNA against P2X_4_ [305, 306]. It has been reported that HIV binding to macrophages stimulates the release of ATP and that P2X_1_ is necessary for the entry of HIV in macrophages [307]. P2Y receptors are also expressed and functional. Low concentrations of ATP were shown to activate PLC and IL-6 transcription [308]. Studies of P2Y_2_ and P2Y_4_ receptor knockout mice led to the conclusion that P2Y_2_ receptors are the dominant P2Y receptor subtype in mouse peritoneal macrophages [309]. Nucleotides, released by apoptotic cells, through pannexin 1 [310], act as ‘find-me’ signals to promote P2Y_2_-dependent recruitment of phagocytic macrophages (as well as monocytes and dendritic cells (DCs)) and this recruitment is reduced in P2Y_2_-deficient mice [3]. The chemoattractant effect of C5a on macrophages was amplified by the release of ATP and the autocrine stimulation of P2Y_2_ and also P2Y_12_ receptors [311]. P2Y_2_ receptors also mediate potentiation of prostaglandin E_2_ release involved in the induction of NOS [312, 313] and stimulate the production of monocyte chemoattractant protein-1 (MCP-1)/chemokine (C-C motif) ligand 2 (CCL2) [314]. Furthermore LPS potentiated nucleotide-induced inflammatory gene expression via upregulation of P2Y_2_ receptors [315]. P2Y_6_ receptor expression also increased following macrophage activation [309]. Indeed the amount of IL-6 and macrophage inflammatory protein-2 released in response to LPS was significantly enhanced in the presence of UDP, and this effect was lost in the macrophages of P2Y_6_ knockout mice [316]. Activation of P2Y_6_ receptors increased the clearance of *Escherichia coli* and improved survival to peritonitis through the release of MCP-1 and enhancement of macrophage chemotaxis [317]. The P2Y_11_ receptor was also reported to be functional in macrophages [318]. These authors observed that ATP released from LPS-activated macrophages by vesicular exocytosis activated the P2Y_11_ receptor, leading to a M1 polarisation characterized by an increased production of IL-12 [318].

#### Microglia

##### P1 receptors

The first evidence of a role of adenosine and its receptors in microglia was derived from the observation of effects of propentophylline, a neuroprotective xanthine derivative that increases the extracellular concentration of adenosine by inhibiting its transport into cells [319]. Propentofylline was shown to inhibit the production of ROS by microglial cells [320, 321], their uptake of amyloid precursor protein [322, 323] and their proliferation and release of TNF-α [324]. Further studies showed that microglia express all subtypes of adenosine receptors. Enhanced activation of microglia associated with worsened demyelination and axonal damage was observed in A_1_ receptor knockout mice subjected to experimental allergic encephalomyelitis [325]. ATP-triggered migration of microglia was inhibited in A_1_^−/−^ as well as CD39^−/−^ mice [326]. The A_3_ receptor is also involved in microglial process extension and migration [327]. On the other hand, ATP acted as a repellent for LPS-treated microglia and induced process retraction; these actions were associated with the upregulation of A_2A_ receptors [328]. A_2A_ receptor knockout mice also displayed enhanced microglial activation in a model of experimental autoimmune encephalomyelitis (EAE) [329].

##### P2 receptors

It was initially reported that ATP, but not ADP, induced an inward current in microglia [330], associated with an increase in cytosolic Ca^2+^ [331]. Further pharmacological studies suggested that these responses were mediated by P2Y receptors [332, 333]. It was later shown that the ATP effect on Ca^2+^ influx was mimicked by BzATP and inhibited by oxATP, supporting the role of the P_2z_ or P2X_7_ receptor [334]. This receptor was shown to mediate the secretion of IL-1β induced by ATP or by LPS via the release of ATP [335], and to induce microglia cell death [336] as well as microglia-mediated injury of neurons [337]. The P2X_7_ receptor was also shown to be involved in microglial activation by amyloid β [338]. After nerve injury, the P2X_4_ receptor was upregulated in the spinal cord and selectively expressed in microglia [339]. The tactile allodynia induced by nerve injury was suppressed by antisense oligodeoxynucleotides silencing P2X_4_ receptors. Knockdown of the P2X_4_ receptor by siRNA inhibited migration of microglia [340].

**Fig. 5:**
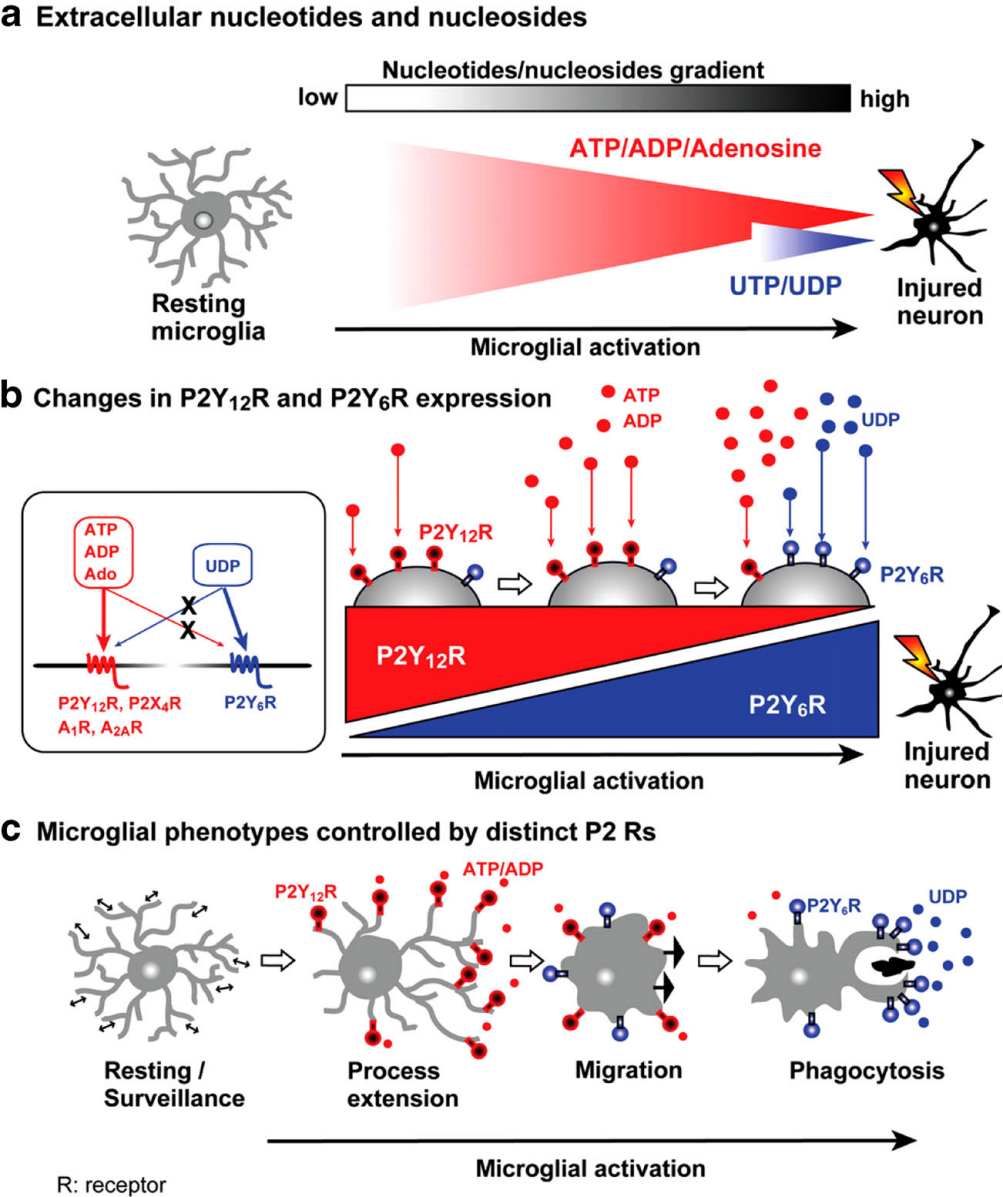
Independence of P2Y_12_ receptor-mediated migration and P2Y_6_ receptor-mediated phagocytosis in microglia. **a** Release/leakage of adenine nucleotides/nucleosides and uridine nucleotides from injured neurons. When neurons or cells are injured or dead, high concentrations of ATP (∼mM) and UTP at a concentration of less than 10 % are leaked. Compared with ATP/ADP/adenosine, UTP/UDP should be transient and localized signals. **b** Changes in P2Y_12_ and P2Y_6_ receptors in microglia according to their activation stages. *Insert* shows pharmacological characterization of P2Y_6_ receptor. UDP is a selective agonist to the P2Y_6_ receptor, and thus, it does not stimulate P2Y_12_, P2X_4_, A_1_, or A_2A_ receptors. Similarly, the P2Y_6_ receptor is a very selective receptor for UDP, and therefore, is not activated by ATP, ADP, or adenosine (Ado). Resting microglia express no or only faint P2Y_6_ receptors; whereas, they express P2Y_12_ receptors adequately. When microglia are activated, they increase P2Y_6_ receptors; whereas, they decrease P2Y_12_ receptors. Only when activated microglia meet UDP at the injured sites do they sense UDP as an eat-me signal. **c** Microglial migration and phagocytosis are controlled by distinct P2 receptors. When microglia sense ATP/ADP by P2Y_12_ receptors, they extrude their processes, followed by migration toward the injured sites. These microglial motilities are not affected by UDP/P2Y_6_ receptors. When activated, microglia upregulate P2Y_6_ receptors, and if they sense the eat-me signal UDP, they start to phagocytose the dead cells or debris. The phagocytic responses are not affected by the activation of P2Y_12_ receptors nor by other P2 or P1 receptors. (Reproduced from [344], with permission from Springer)

Following brain injury, microglia extrude processes and migrate toward sites of tissue damage. Polarisation, process extension and chemotaxis did not occur in P2Y_12_-deficient mice, while baseline motility was normal [341]. Furthermore, in living P2Y_12_-deficient mice, branch extension toward sites of cortical damage was decreased. Microglial activation leads to the downregulation of P2Y_12_ receptors and the upregulation of P2Y_6_ receptors [342, 343]. Activation of P2Y_6_ receptors by UDP stimulates phagocytosis and the uptake of microspheres. *In vivo* an upregulation of P2Y_6_ was observed following administration of kainic that damages neurons, leading to microglia activation. Taken together these findings show that ADP, acting through P2Y_12_, is a find-me signal for microglia, whereas UDP, acting on P2Y_6_, behaves as an eat-me signal [344] (Fig. 5).

#### Section summary

ATP released from apoptotic cells constitutes a find-me signal that attracts monocytes/macrophages, an action mediated by the P2Y_2_ receptor. It stimulates bacterial killing and macrophage apoptosis thereby contributing to decrease the bacterial and parasite burden: this action is mediated by the P2X_7_ receptor. ATP also exerts a proinflammatory effect through the secretion of IL1-β, which is mediated by the P2X_7_ receptor and NLRP3 inflammasome.

In contrast, adenosine exerts an inhibitory effect on monocytes/macrophages mediated by A_2A_ and A_2B_ receptors.

Multiple P1 and P2 receptors have been shown to play a role in microglia. The P2X_7_ receptor is involved in IL-1β secretion and cell death. P2Y_12_, P2X_4_, A_1_ and A_3_ receptors stimulate process extension and migration, whereas the A_2A_ receptor is inhibitory. On the other hand the P2Y_6_ receptor is upregulated in activated microglia and triggers microglial phagocytosis.

### Dendritic cells

#### P1 receptors

CD39 and CD73 ectonucleotidases [345] as well as A_1_, A_2A_ and A_3_ but not A_2B_ receptors [346] are expressed by human monocyte-derived DCs. In immature DCs, adenosine induced calcium transients but no increase in cAMP. This resulted in actin polymerization, chemotaxis [346] and increased expression of co-stimulatory molecules [347]. Maturation of DCs by LPS resulted in downregulation of A_1_ and A_3_ receptor mRNA, whereas A_2A_ receptors were still expressed [346]. In these mature DCs, adenosine increased cAMP and inhibited IL-12 and TNF-α production, whereas it enhanced IL-10 secretion [346, 347]. These results show that adenosine can act as a chemotaxin for immature human DCs and induce their semi-maturation, characterized by a reduced capacity to induce a Th1 polarisation of CD4^+^ T lymphocytes [347]. Adenosine via cAMP also decreased the capacity of human DCs to prime CD8^+^ T cells [348].

In murine monocyte-derived DCs, adenosine also impaired maturation and inhibited the production of IL-12, leading to tolerance: this effect was mediated by the A_2B_ receptor instead of the A_2A_ receptor active in human cells [349–351]. IL-27 is a cytokine produced by DCs that suppresses Th1 and Th17 responses and limits inflammation in several experimental models. The suppressive action of IL-27 was mediated at least in part by the induction of CD39 in DCs and the resulting accumulation of adenosine [352]. However, the observation that adenosine could also promote the development of murine Th17 cells, via the A_2B_ receptor- mediated production of IL-6, added an additional complexity [353]. The A_2B_ receptor was upregulated in EAE and A_2B_ knockout mice developed less severe EAE than wild-type mice [354]. In human plasmacytoid DCs, adenosine plays a dual role by initially recruiting immature cells to sites of inflammation, an effect mediated by A_1_ receptors, and by subsequently inhibiting the production of IL-6 and IFN-α, via the A_2A_ receptor [355].

The physiological importance of the inhibitory effect of adenosine on DCs is supported by observations on the role of ADA. Indeed the high ADA activity of DCs might help to maintain them in an active state [356]. ADA has been shown to be upregulated in DCs from non-obese diabetic (NOD) mice leading to their spontaneous activation and autoimmune T cell activation [357]. Paradoxically DCs from CD39^−/−^ mice exhibited impaired antigen-presenting capacity and ability to induce a Th2 response [358, 359]. This resulted in decreased allergic contact hypersensitivity [358] and allergic airway inflammation [359]. This was explained not by a defect in adenosine formation but by an increased accumulation of ATP leading to the desensitization of P2Y receptors (see below).

Finally it must be mentioned that inosine has been reported to induce DCs chemotaxis independently from adenosine receptors [360]. On the other hand AMP was shown to mimic the inhibitory effects of adenosine on DCs, and these effects were maintained in CD73-deficient mice and could not be explained by adenosine contamination of AMP [361]. The mechanisms of these effects remain unknown.

#### P2 receptors

Human DCs express mRNA for almost all known P2 receptors [345, 362–364] and extracellular nucleotides exert multiple effects on them ranging from chemotaxis to control of cytokine release and induction of cell death. P2Y but not P2X agonists are potent chemotactic stimuli for immature but not mature DCs [364]. Chemotaxis was associated with a rise in intracellular Ca^2+^ and actin polymerization and involved the activation of G_i_. Allergen challenge was shown to cause acute accumulation of ATP in the airways of asthmatic subjects and mice with experimentally induced asthma that resulted in the recruitment of DCs [1]. That recruitment was mediated by the P2Y_2_ receptor. Indeed, *in vitro* the ATP-induced migration of P2Y_2_-deficient DCs was strongly decreased as compared to DCs from wild-type mice [115]. The attraction of DCs to the lungs in a model of allergic inflammation induced by ovalbumin was also decreased in P2Y_2_^−/−^ mice [115]. Decreased attraction of DCs to the airways might also explain the higher mortality of P2Y_2_^−/−^ mice with lung infection by pneumonia virus of mice, as a consequence of lowered immune response and viral clearance [365]. Interestingly, the formation of ATP gradients at a site of inflammation can also inhibit transiently the migration of human DCs, via the P2Y_11_ receptor, and thereby prolong the time of encounter with antigens [366]. Conversely, antagonism of the P2Y_11_ receptor might improve the migration of antigen-loaded DCs to the lymph nodes. In addition to these direct effects on migration, ATP modulated the expression of chemokine receptors, with an induction of CXCR4 and a reduction of CCR5 [367], and inhibited the release of CCL2 and CCL3 chemokines [368].

Nucleotides were also shown to modulate the maturation of DCs. Schnurr et al. [369] initially reported that ATP stimulates the expression of CD83 and the secretion of IL-12 by human monocyte-derived DCs. This action was shown to be mediated by the P2Y_11_ receptor and a rise in cAMP [370]. However, la Sala et al. [371] confirmed that ATP stimulates the maturation of DCs but observed an inhibitory effect on the release of IL-12 stimulated by LPS, leading to an impaired ability to initiate Th1 responses. These apparent discrepancies were resolved by the demonstration that ATP, via P2Y_11_, increased IL-12p40 but inhibited the production of IL-12p70 [372]. Furthermore ATP synergized with LPS and sCD40L to stimulate IL-10 production. This led to the conclusion that ATP, via the P2Y_11_ receptor, induces a semi-maturation of DCs, characterized by an increased expression of co-stimulatory molecules and a decreased production of bioactive IL-12, leading to increased Th2 responses or tolerance. Additional studies showed that ATP via the P2Y_11_ receptor produced an impressive upregulation of the expression of thrombospondin-1 and indoleamine 2,3-dioxygenase that could play a major role in tolerance [373]. A systematic study of the effect of ATP on gene expression in DCs revealed a P2Y_11_-mediated stimulatory effect on the expression of VEGF-A, that has immunosuppressive effects in addition to its angiogenic action [374], and amphiregulin, that can exert an angiogenic and tumorigenic action [375].

Other P2Y receptors were found to be expressed on monocyte-derived DCs. ATP can modulate the function of DCs directly via a cAMP increase mediated by P2Y_11_ receptors and indirectly via its degradation into ADP, which acts on P2Y_1_ receptors; these distinct mechanisms combine to inhibit inflammatory cytokine production, particularly IL-12, but have a differential effect on IL-10 [376]. P2Y_12_ receptors are also expressed by murine DCs and their activation increased antigen endocytosis with subsequent enhancement of T cell activation [377]. UDP, but not UTP, stimulated the release of CXC-chemokine 8 from mature human DCs, via P2Y_6_ receptors [378]. UTP and UDP also acted on murine DCs to mobilize intracellular Ca^2+^ and to induce cytokine production [379]. Human immature monocyte-derived DCs express P2Y_14_ receptors that mediate an increase in [Ca^2+^]_i_ in response to agonists [380]. In plasmacytoid DCs, UTP, UDP and UDP-glucose were shown to inhibit IFN-α production [381].

Like in macrophages, ATP induced in DCs the NLRP3/ASC inflammasome signalling complexes that drive proteolytic maturation and secretion of the proinflammatory cytokines IL-1β and IL-18 [382, 383]. This action was mediated by the P2X_7_ receptor, which is functionally expressed on DCs [384]. P2X_7_ receptors were shown to be present in microvesicles shed from DCs together with IL-1β and caspase-1 and caspase-3 [385]. P2X_7_-deficient DCs fail to release IL-1β in response to LPS and ATP [386]. This might explain the resistance to allergic contact dermatitis observed in P2X_7_-deficient mice [386]. Additional P2X_7_-mediated effects of ATP on DCs include shedding of CD23 [387], release of tissue factor-bearing microparticles [388] and apoptosis [389, 390].

In the intestine ATP released from commensal bacteria induced the differentiation of Th17 CD4^+^ cells via the activation of lamina propria CD11c^+^ antigen-presenting cells, apparently via a P2X receptor [391]. The number of Th17 cells was increased in mice deficient in ENTPDase7, which is preferentially expressed on epithelial cells of the small intestine [392].

#### Section summary

ATP can exert multiple actions on DCs, mediated by distinct receptors: chemotaxis mediated mainly by the P2Y_2_ receptor; semi-maturation, characterized by increased expression of co-stimulatory molecules and inhibition by IL-12, which is mediated by the P2Y_11_ receptor and associated with a Th2 response or tolerance; induction of NLRP3/ASC inflammasome signalling complexes, mediated by the P2X_7_ receptor, that leads to secretion of IL-1β and a proinflammatory effect; and enhanced antigen endocytosis mediated by the P2Y_12_ receptor.

Adenosine acting on the A_2A_ or A_2B_ receptor exerts complex effects on DCs: as ATP it impairs Th1 polarisation and favours Th2 and/or tolerance, but it can also favour Th17 cell development.

### Lymphocytes

#### T and B lymphocytes

##### P1 receptors

Adenosine was reported to cause an increase in cAMP in lymphocytes as well as in thymocytes [393–396] and to have powerful inhibitory effects on lymphocyte proliferation [397] and the immune response in humans, particularly those who have inherited deficiency of ADA [398]. The destruction of tumour cells by mouse lymphocytes was shown to be inhibited by adenosine, and this effect was potentiated by an inhibitor of ADA [399]. It was suggested that this effect of adenosine may contribute to the lack of immune response associated with ADA deficiency.

ATPase, ADPase, 5′-nucleotidase and ADA have been shown to be present on human lymphocytes [400–403]. Although it was claimed that adenosine release results from the intracellular degradation of ATP to adenosine, later studies showed that extracellular adenosine is generated following the release of ATP and its extracellular breakdown [404, 405]. Human B lymphocytes showed high degrading activity, while T lymphocytes were reported to be unable to degrade extracellular nucleotides [404]. However, ecto-ATPase activity was reported on cytolytic T lymphocytes [406], and E-NTPDase activity was upregulated within 15 min of T cell stimulation [407]. Furthermore, a subset of T regulatory (Treg) cells expresses CD39 and CD73 ectonucleotidases (see below). However it was suggested that CD39 is not the exclusive switch of the immune system to trigger immunosuppression, and that an alternative adenosine-generating axis is operating [408]. This axis involves the enzymes CD38 (a nicotinamide adenine dinucleotide (NAD^+^) nucleosidase) and CD303a (an ecto nucleotide pyrophosphatase).

A_2A_ receptors were shown to be expressed on T lymphocytes [409–411]. A_2B_ receptors were also shown to be expressed on human T lymphocytes, and it was suggested that they play a role in lymphocyte deactivation by adenosine [412]. In another study, it was suggested that A_2A_ receptors vary in their expression on T cell functional subsets and may regulate cytokine production in activated T lymphocytes [413]. There was lower expression of A_2A_ receptors on B cells. A_3_ receptor mRNA and protein were shown to be expressed in both resting and activated human lymphocytes and under activating conditions they are upregulated [414]. Stimulation of A_1_ and A_3_ receptors were reported to block the inhibitory action mediated by A_2A_ receptors [415]. Exposure to adenosine prior to antigenic stimulation also induced a desensitization of cAMP accumulation leading to a stronger response to antigenic stimulation [416].

Conclusive evidence for the major role of A_2A_ receptor in the regulation of T lymphocytes came out of the study of A_2A_-deficient mice. cAMP accumulation in response to adenosine was decreased in T cells from A_2A_^+/−^ mice and almost abolished in those of A_2A_^−/−^ mice [417]. In CD4^+^ T cells, a selective A_2A_ agonist had a major inhibitory effect on the T cell receptor (TCR)-mediated production of IFN-γ and this effect was decreased by 50 % in cells of A_2A_^+/−^ mice and completely abolished in those from A_2A_^−/−^ mice [418]. A_2A_ receptor activation inhibited T cell proliferation and IL-2 production whether the cells were expanded under Th1 or Th2-skewing conditions, and again this inhibition was abolished in A_2A_-deficient mice [419]. Furthermore, TCR stimulation caused a rapid increase in A_2A_ mRNA, both in Th1 and Th2 cells [418, 419].

Adenosine via A_2A_ receptors exerts other effects on T cells. The apoptotic effect of adenosine on resting T cells was inhibited in A_2A_^−/−^ mice [417, 420]. On the other hand, adenosine via A_2A_ receptor inhibited activation-induced cell death of already activated T cells [421]. Furthermore, the A_2A_ receptor contributes to the maintenance of a normal number of naive T cells by inhibiting TCR-induced activation [422]. Adenosine also inhibits T cell mobility [423], migration to lymph nodes [424] and adhesion to the endothelium [425].

**Fig. 6:**
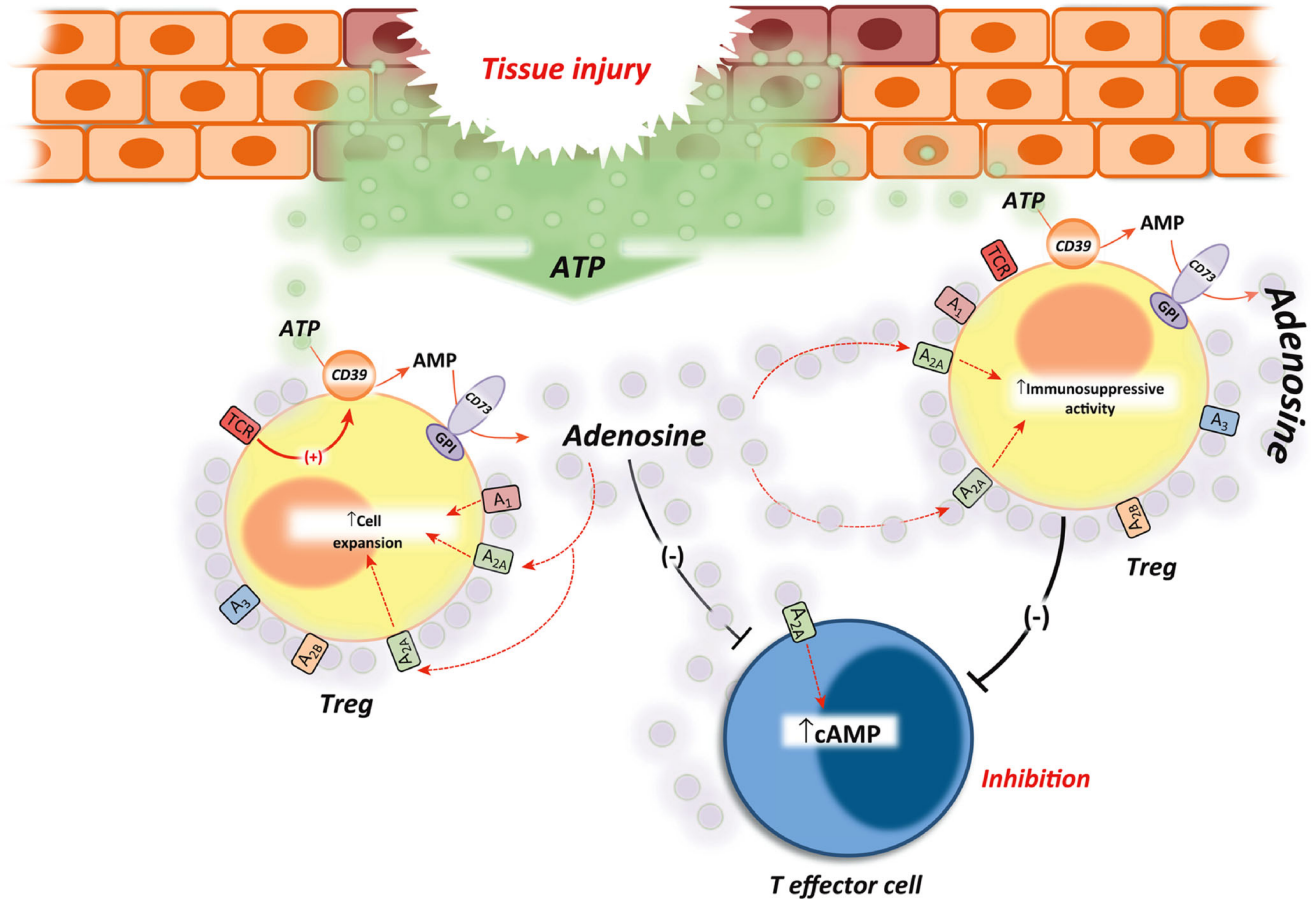
The CD39/CD73 pathway modulates regulatory T cell (*Treg*) activity. The activation of T cell receptor (*TCR*), expressed on Tregs, induces CD39 activity. This increment of ATP-metabolizing activity is critical for the immunosuppressive activity of Tregs because it facilitates the pericellular generation of adenosine, a substantial component of the immunosuppressive and anti-inflammatory functions of Tregs. The inhibitory action of Treg-derived adenosine can be ascribed to the activation of A_2A_ receptors expressed on T effector cells, which undergo reduced immune activity. In addition, adenosine generation triggers a self-reinforcing loop of Treg functions because the stimulation of A_2A_ receptors expressed on these cells elicits their expansion and increases their immunoregulatory activity. (Reproduced from [426], with permission from Elsevier)

The importance of the A_2A_-mediated inhibitory effect of adenosine on T cells was underscored by the discovery that CD39 is selectively expressed on Treg cells (see Fig. 6) [426] that are essential for maintaining peripheral tolerance [427, 428]. In human T cells, CD39 is expressed primarily by immunosuppressive Treg cells that express the Foxp3 transcription factor, and its activity is enhanced by TCR ligation [429]. CD73 is also expressed on CD4^+^/CD25^+^/Foxp3 Treg cells [430–432]. However, subsets of Treg cells expressing CD39, but not CD73, have been identified [433]. Inhibition of ADA activity further enhanced Treg-mediated immunosuppression [432].

Several studies have shown the impact of adenosine-mediated inhibition of T cells in various models of disease. Defective adenosine-induced cAMP accumulation and immunosuppression were reported in T lymphocytes of patients with systemic lupus erythematosus [434, 435]. A_2A_ receptor activation during reperfusion after ischemia protected the myocardium from infarction and this effect was dependent on an inhibition of T cell accumulation [436, 437]. A_2A_ receptor agonists attenuated allograft rejection and alloantigen recognition by an action on T lymphocytes [438], suppressed the development of graft-versus-host disease [439, 440] and attenuated experimental autoimmune myasthenia gravis [441]. CD39 and CD73 expressed on Treg cells led to a local accumulation of adenosine that protected against Helicobacter induced gastritis [442]. Treg cells suppressed contact hypersensitivity reactions by a CD39 and adenosine-dependent mechanism [425]. In other models, the action of adenosine proved to be deleterious. A_2A_-deficient mice were protected from the lethal effect of sepsis, due to preserved lymphocyte function and decreased immunosuppressive IL-10 [443]. CD39 and CD73 expressed on ovarian cancer cells generate adenosine that exerts an immunosuppressive effect, which was relieved by siRNAs against CD39 and CD73 and by an A_2A_ antagonist [444]. In HIV infection, Treg inhibitory effects were relieved by CD39 downregulation and reproduced by an A_2A_ agonist [445, 446]. Furthermore, a polymorphism of the CD39 gene was identified, that is associated with downregulation of CD39 and slower progression to AIDS [446].

Few studies have been performed on B lymphocytes. Accumulation of cAMP produced by adenosine in B cells stimulated by *Staphyloccocus aureus* suppressed IgM production [447]. On the other hand B cells coexpress CD39 and CD73 and adenosine inhibited B cell proliferation and cytokine expression [448]. Activated B cells also inhibited T cell proliferation and cytokine production [448].

##### P2 receptors

Early reports showed that ATP protected rat lymphocytes against the loss of intracellular enzymes into the medium [449, 450] and that receptors for ATP were present on lymphocytes [451]. The action of ATP on lymphocytes is complex: ATP was reported to stimulate DNA synthesis in a subpopulations of T cells [452, 453], but ATP was also shown to be highly toxic to human lymphocytes and to thymocytes, causing permeabilization of the plasma membrane and cell death [454, 455]. It was later shown that ATP increased cytosolic Ca^2+^ in mouse thymocytes [456–458] and stimulated the PLC pathway in human B lymphocytes [459]. On the other hand, an ATP^4−^ receptor-operated sodium channel was identified on human lymphocytes [460, 461]. ATP-gated channels were also identified in human lymphoblasts [462].

**Fig. 7:**
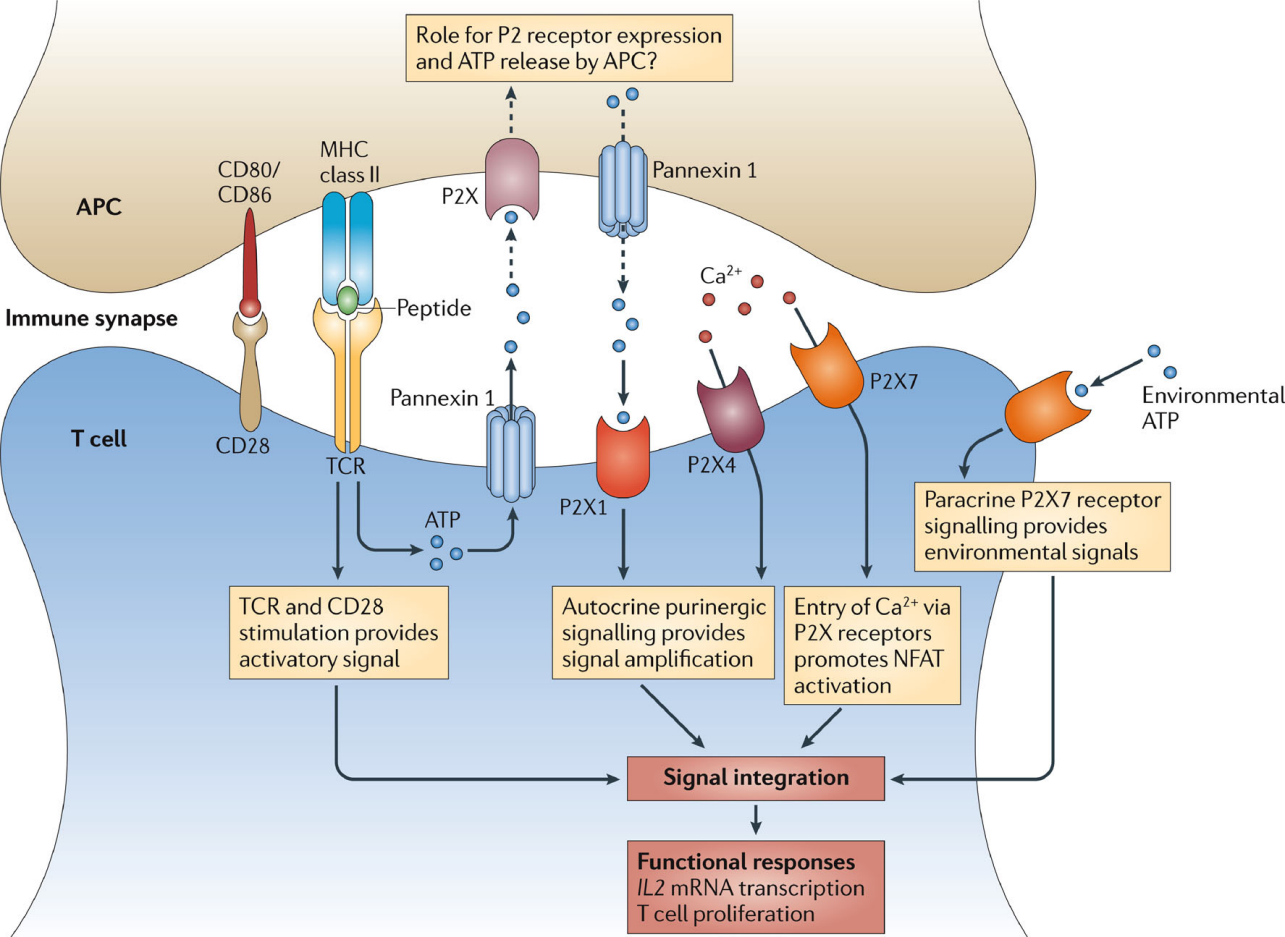
Purinergic signalling in T cell activation. Antigen recognition by T cells involves the formation of an immune synapse between a T cell and an antigen-presenting cell (*APC*). The immune synapse contains a large number of signalling molecules that are required for T cell activation, including T cell receptors (*TCRs*), MHC molecules, co-stimulatory receptors and the purinergic signalling receptors P2X_1_, P2X_4_ and P2X_7_. In response to TCR and CD28 stimulation, pannexin 1, P2X_1_ receptors and P2X_4_ receptors translocate to the immune synapse. ATP released through pannexin 1 promotes autocrine signalling via the P2X receptors. Confinement of ATP in the immune synapse results in a powerful autocrine feedback mechanism that facilitates the signal amplification required for antigen recognition. P2 receptors expressed and ATP released by APCs may also have important roles in regulating the antigen recognition process. *NFAT* nuclear factor of activated T cells. (Reproduced from [474], with permission from Springer)

Important advances were made in 1994 and the following years with the identification on human lymphocytes of P_2Z_, now called P2X_7_, receptors antagonised by oxATP [463] and by the isoquinoline derivative KN-62 [464]. P2X_7_ receptors were also identified specifically in human B lymphocytes [465, 466] and murine T lymphocytes [467]. P2X_7_ receptors were implicated in the mitogenic stimulation of human T lymphocytes purified from peripheral blood [468]. ATP and the selective P2X_7_ agonist BzATP caused plasma membrane depolarisation and a Ca^2+^ influx in T lymphocytes. ATP or BzATP alone had no effect on lymphocyte proliferation but potentiated the action of mitogens such as anti-CD3 [468]. Transfection of lymphoid cells lacking P2X_7_ receptors with P2X_7_ cDNA increased their proliferation [469]. Later studies showed that TCR stimulation triggers the release of ATP through pannexin-1 hemichannels [470] and vesicular exocytosis [471], and upregulates P2X_7_ expression [472]. siRNA silencing of P2X_7_ inhibited T cell activation, which was also lower in C57BL/6 mice that express a poorly functional P2X_7_ receptor, as compared to BALB/c mice that express fully functional P2X_7_ receptors [472]. Shockwaves increased T cell proliferation through ATP release and P2X_7_ activation [473]. Thus ATP released through pannexin 1 channels enhances T call activation in an autocrine manner (Fig. 7; [474]). But it is also involved in a paracrine communication that leads to calcium waves in neighbouring lymphocytes and a reduction of T cell motility in lymph nodes that would favour T cell scanning of antigen-loaded DCs [475]. However, P2X_7_ receptors also induced the shedding of L-selectin (CD62L) from T cells, which accompanies T cell activation and allows T cells to move away from lymph nodes and enter the circulation [476–482].

ATP induced the lysis of CD4^+^ thymocytes and peripheral CD4^+^ T cells [483] and the apoptosis of murine thymocytes [484, 485]. T lymphocyte subsets express different levels of P2X_7_ and high levels are associated with ATP-induced cell death [486]. P2X_7_ receptor-mediated cell death was also shown to differ between different stages of murine T cell maturation [487]. Interestingly mouse Treg cells express a higher level of P2X_7_ and their activation by ATP leads to their depletion [488, 489]. P2X_7_^−/−^ mice have increased levels of Treg cells [490]. The P2X_7_ receptor was also involved in T cell death induced by NAD^+^ through the ADP-ribosylating ectoenzyme, ART2. Indeed ART2-catalyzed ADP-ribosylation activates P2X_7_ receptors [491–493]. In particular Treg cells express ART2 and can be depleted by intravenous injection of NAD^+^ [494]. However, ATP (1 mM) enhanced the proliferation and immunosuppressive ability of human Treg cells, whereas it induced apoptosis of CD4^+^ T cells [495]. The dual action of the P2X_7_ receptor on growth versus death clearly depends on the concentration of ATP, with stimulatory effects at 250 nM and inhibition at 1 mM [495]. This could be related to the existence of two states of activation of the P2X_7_ receptor: cation-selective channel or large conductance non-selective pore [496].

Numerous studies have shown the importance of the lymphocyte P2X_7_ receptor in various models of inflammatory diseases. In some of these models, inhibition or deficiency of P2X_7_ was associated with decreased immune reactions. *Mycobacterium tuberculosis* infected P2X_7_^−/−^ mice had an increased microbial burden in the lung and pulmonary infiltrates contained a higher number of Treg cells [497]. oxATP was shown to inhibit T cell-mediated autoimmunity in models of autoimmune type 1 diabetes and encephalitis in mice [498]. CD38 knockout NOD mice develop accelerated type 1 diabetes. This was corrected by coablation of P2X_7_ [499]. oxATP delayed islet allograft rejection [500] and increased cardiac transplant survival in mice [501]; these effects were associated with decreased T cell activation. However in other models P2X_7_ deficiency was associated with increased immune reactions, illustrating the dual role of P2X_7_ receptors emphasized previously. Following oral infection with *Listeria monocytogenes*, P2X_7_-deficient mice showed enhanced CD8 responses in the intestinal mucosa, which can be explained by the proapoptotic effect of P2X_7_ on intestinal CD8 cells [502]. Graft versus host disease was enhanced in P2X_7_^−/−^ mice, and this is associated with T cell expansion and reduced Treg cells [503]. EAE was also exacerbated in P2X_7_^−/−^ mice as a result of decreased apoptosis of T lymphocytes [504] and increased T cell cytokine production [505].

 P2X receptors other than P2X_7_ have been shown to play a role in T cell control. RT-PCR studies had shown that P2X_1_, P2X_2_ and P2X_6_ were expressed by murine thymocytes in addition to P2X_7_ [506]. ATP released through pannexin hemichannels following TCR stimulation amplified T cell activation not only through P2X_7_ receptors [472] but also via P2X_1_ and P2X_4_ receptors, as demonstrated by the use of siRNA [474, 507]. Hypertonic saline is known to increase T cell function [508]: it acts through the release of ATP and the activation of P2X_1_, P2X_4_ and P2X_7_ receptors, as shown also by gene silencing [509]. Both P2X_7_ and P2X_4_ are also involved in the activation of unconventional βγ T cells [510, 511].

Although P2X receptors and particularly P2X_7_ play a major role in lymphocytes, there is some evidence for the role of P2Y receptors as well. Upregulation of P2Y_2_ receptor mRNA expression was described as an immediate early gene response in activated thymocytes [512] and P2Y_2_ receptors were shown to be involved in ATP-induced T cell migration [495]. The P2Y_6_ receptor was shown to be expressed in activated T cells infiltrating in inflammatory bowel disease [513]. Antagonists of the P2Y_6_ receptor blocked murine T cell activation [514], but these results must be interpreted with caution since T cells of P2Y_6_-deficient mice exhibited an increased activity in a model of allergic pulmonary inflammation, suggesting that the P2Y_6_ receptor plays an inhibitory rather than a stimulatory role [515]. P2Y_14_ receptors were shown to be functionally expressed by mouse spleen-derived T lymphocytes [516]. Adenine nucleotides inhibited CD4^+^ T cell activation via an increase in cAMP induced by an unidentified P2Y receptor [517].

#### Natural killer (NK and NKT) cells

##### P1 receptors

NK cell activity was shown to be inhibited by adenosine and A_2_ receptor agonists that increase cAMP [518]. Later studies demonstrated the involvement of A_2A_ receptors [519]. Adenosine via the A_2A_ receptor inhibited IFN-γ production by NKT cells, a subset of T cells with natural killer activity [520], but increased their production of IL-4 and IL-10 [521]. Mice were protected against liver reperfusion injury and concanavalin A (ConA)-induced hepatitis by adenosine acting on the A_2A_ receptor on NKT cells, and this protection was abolished in A_2A_^−/−^ mice [520, 522]. Sickle cell disease results in disseminated microvascular ischemia and reperfusion injury that leads to the activation of NKT cells and the upregulation of A_2A_ receptors [523–525]. Activation of A_2A_ receptors in NY1DD mice with sickle cell disease reduced pulmonary inflammation and injury [523]. In a phase I study, the A_2A_ agonist regadenoson was administered to patients with sickle cell disease and was shown to inhibit the activation of NKT cells [524]. A_2A_^−/−^ mice were protected against tumor metastasis, and this protection was associated with increased NK cell maturation and cytotoxic function [526]. On the other hand, an A_3_ receptor agonist was shown to potentiate NK cell cytotoxic activity [527] and IFN-γ production [528].

##### P2 receptors

Inhibition of human and mouse NK cell reactivity via nucleotide receptors was reported [529–532]. It was later shown that ATP inhibits cell killing by NK cells via the P2Y_11_ receptor and an increase in cAMP [533]. On the other hand, NKT cells express the P2X_7_ receptor, the activation of which can lead to either apoptosis or cell activation [534–536]. *In vitro* NAD induced rapid apoptosis of NKT cells that was mediated by the P2X_7_ receptor, but its injection in Con A-treated mice enhanced cytokine production by NK cells and liver injury, that was decreased in P2X_7_ knockout mice [534]. In CD39-deficient mice, apoptosis of NKT cells was increased leading to protection against ConA-induced liver injury [535] or hyperoxic lung injury [536].

#### Section summary

The release of ATP through pannexin hemichannels or vesicular exocytosis amplifies in an autocrine way the TCR-mediated activation of T lymphocytes. This amplification is mediated by the P2X_7_ receptor, and also by P2X_1_ and P2X_4_ receptors. But activation of P2X_7_ can also induce T cell death. The resulting effect (activation or death) depends on the particular subset of T cells and on the concentration of ATP.

Adenosine exerts inhibitory effects on T lymphocytes, which are mediated by the A_2A_ receptor. Treg cells over-express the ectonucleotidases CD39 and CD73 that sequentially convert ATP into AMP and adenosine, and their immunosuppressive action is partially mediated by adenosine.

## Concluding remarks

Extracellular nucleotides and adenosine exert a variety of effects on distinct subsets of immune cells via a wide spectrum of receptor subtypes (Table 2). These actions can be both stimulatory and inhibitory, and the balance between the two critically depends on the amount and time course of nucleotide release. This is consistent with the role of ATP and its degradation product adenosine as danger signals that stimulate the immune response following injury but moderate this response when it becomes excessive and deleterious.

**Table 2:** Expression profiles and functional responses of the purinergic receptor subtypes in different immune cells

Inflammatory cell type	Functional response to purines	P2 receptor subtype (expression profile and/or involvement in functional response)
Neutrophils	Undefined roles	P2Y_1_, P2Y_4_, P2Y_11_, P2Y_14_ and P2X_7_
	Calcium mobilization	P2Y_2_
	Actin polymerization	P2Y_2_
	Primary granule release	P2Y_2_
	Chemotaxis	P2Y_2_, P2Y_6_ and P2X_1_
	Reduced cAMP accumulation	P2Y_14_
	Delay in constitutive neutrophil apoptosis	P2Y_11_
Macrophages	Undefined roles	P2Y_4_, P2Y_6_, P2Y_11_, P2Y_12_, P2Y_13_, P2Y_14_ and P2X_1_–P2X_6_
	Intracellular calcium increase	P2Y_1_, P2Y_2_, P2Y_4_, P2Y_11_, P2X_4_ and P2X_7_
	IL-1β/IL-18 maturation/release via the NLRP3 inflammasome, caspase-1 and cytosolic K^+^ depletion	P2X_7_
	Release of cathepins, PGE_2_, MMP-9 phosphatidilserine (caspase independent)	P2X_7_
	Promoting chemotaxis/phagocytosis	P2Y_2_, P2Y_12_, P2X_1_ and P2X_3_
	Regulation of autophagy	P2X_4_ and P2X_7_
	Multinucleated giant cells formation	P2X_7_
Dendritic cells	Undefined roles	P2X_1_, P2X_4_, P2X_5_, P2X_7_, P2Y_1_, P2Y_4_, P2Y_6_ and P2Y_11_
	Regulation in cytokine release	P2Y_11_
	DC maturation	P2Y_11_, P2Y_12_ and P2Y_14_
	Apoptosis	P2X_7_
	DC migration	P2Y_2_ and P2Y_11_
Lymphocytes		
B and T cells	Undefined roles	P2X_2_, P2X_3_, P2X_5_, P2X_6_ and all P2Y
	T cell activation (p38 MAPK activation and IL-2 gene transcription )	P2X_1_, P2X_4_ and P2X_7_
	T cell activation (CD62L shedding)	P2X_7_
	cAMP accumulation	P2Y_14_
	Inhibition of immunosuppressive potential of Tregs	P2X_7_
Natural killer cells	Regulation of NK cytoxicity and chemotaxis	P2Y_11_
Eosinophils	Undefined roles	P2Y_1_, P2Y_4_, P2Y_6_, P2Y_11_, P2Y_14_, P2X_1_, P2X_4_ and P2X_7_
	Chemotaxis	P2Y_2_
	Release of chemokines and cytokines	P2Y_2_, P2X_1_, P2X_7_ and P2Y_6_
Mast cells	Undefined roles	P2X_1_, P2X_4_, P2X_6_, P2X_7_, P2Y_1_, P2Y_2_, P2Y_11_, P2Y_12_ and P2Y_13_
	Degranulation	P2Y_13_ and P2Y_14_

Reproduced from [550], with permission from Springer

### Neutrophils and eosinophils

ATP released from neutrophils amplifies their attraction by chemotactic signals via the P2Y_2_ receptor and after its degradation to adenosine via the A_3_ receptor, one example of cooperation between P1 and P2 receptors. The P2Y_2_ receptor is also involved in the recruitment of eosinophils in the lung during allergic inflammation. On the other hand ATP and adenosine have opposite effects on O_2_^−^ production and other functions of neutrophils: potentiation by ATP and inhibition by adenosine.

### Monocytes/macrophages and microglia

ATP released from apoptotic cells constitutes a find-me signal that attracts monocytes and macrophages, an action mediated by the P2Y_2_ receptor. Via the P2X_7_ receptor, ATP stimulates NLRP3 inflammasome activation and IL-1β secretion by macrophages, their killing of bacteria and their apoptosis.

ADP acting on the P2Y_12_ receptor induces the polarisation and migration of microglia. UDP acting on the P2Y_6_ receptor stimulates their phagocytic activity. ADP and UDP have, thus, a complementary action of find-me and eat-me signals, respectively, involving a cooperation between two distinct P2Y receptor subtypes. The P2X_4_, A_1_ and A_3_ receptors have also been shown to play a role in microglia migration, whereas the A_2A_ receptor is inhibitory.

### Dendritic cells

ATP can exert an immunostimulatory effect on DCs via P2X_7_ receptor activation. But it can also activate the P2Y_11_ receptor leading to a semi-maturation state characterized by the upregulation of co-stimulatory molecules and the inhibition of IL-12 production, which impairs the Th1 response and favours tolerance or a Th2 response. The balance between these opposite effects depends on the amount of ATP released and the time course of this release.

Other specific functions of DCs can be activated by nucleotides via distinct P2Y receptor subtypes: chemotaxis by the P2Y_2_ receptor and antigen endocytosis by the P2Y_12_ receptor. Adenosine acting on the A_2A_ (human) or A_2B_ (mouse) receptors exerts complex effects on DCs: as ATP it impairs Th1 polarisation and favours Th2 and/or tolerance, but it also favours Th17 cell development.

### Lymphocytes

The release of ATP through pannexin hemichannels or vesicular exocytosis amplifies in an autocrine way the TCR-mediated activation of T lymphocytes. This amplification is mediated by the P2X_1_, P2X_4_ and P2X_7_ receptors. On the other hand, Treg cells over-express the ectonucleotidases CD39 and CD73 that sequentially convert ATP into AMP and adenosine, which binds to A_2A_ receptors on effector T cells and suppresses their function.

### Neuroimmunology

Contrary to earlier beliefs, the evidence that immune cells are innervated, albeit by nerve varicosities that form occasional close appositions, is convincing. This will be important for future studies of neuroimmunology that might reveal additional roles of ATP and purinergic signalling.
